# Chitosan/Poly(2-ethyl-2-oxazoline) Films with Ciprofloxacin for Application in Vaginal Drug Delivery

**DOI:** 10.3390/ma13071709

**Published:** 2020-04-06

**Authors:** Guzel K. Abilova, Daulet B. Kaldybekov, Galiya S. Irmukhametova, Diara S. Kazybayeva, Zhanar A. Iskakbayeva, Sarkyt E. Kudaibergenov, Vitaliy V. Khutoryanskiy

**Affiliations:** 1 Department of Chemistry and Chemical Technology, Al-Farabi Kazakh National University, Almaty 050040, Kazakhstan; guzelab82@mail.ru (G.K.A.); dauletchem@gmail.com (D.B.K.); galiya.irm@gmail.com (G.S.I.); diara_92@mail.ru (D.S.K.); 2 Department of Natural Sciences, K. Zhubanov Aktobe Regional State University, Aktobe 030000, Kazakhstan; 3 Microbiology Laboratory of the Scientific Center for Anti-Infectious Drugs, Almaty 050060, Kazakhstan; zhanara_07_74@mail.ru; 4 Institute of Polymer Materials and Technologies, Almaty 050019, Kazakhstan; skudai@mail.ru; 5 Reading School of Pharmacy, University of Reading, Whiteknights, Reading RG6 6AD, UK

**Keywords:** chitosan, poly(2-ethyl-2-oxazoline), films, antibacterial activity, mucoadhesion, drug release, vaginal drug delivery

## Abstract

Chitosan (CHI) and chitosan/poly(2-ethyl-2-oxazoline) (CHI/POZ)-based films were prepared by casting from aqueous solutions of polymer blends with different compositions. Ciprofloxacin was used as a model drug in these formulations. The weight, thickness, folding endurance and transparency of blend films were measured and characterised. All films had a uniform thickness (0.06 ± 0.01 mm) and exhibited sufficient flexibility. The surface pHs of films ranged from 3.76 ± 0.49 to 4.14 ± 0.32, which is within the pH range suitable for vaginal applications. The cumulative release of the drug from the films in experiments in vitro was found to be 42 ± 2% and 56 ± 1% for pure CHI and CHI/POZ (40:60) films, respectively. Drug-free chitosan/poly(2-ethyl-2-oxazoline) films showed weak antimicrobial activity against Escherichia coli. Drug-loaded CHI and CHI/POZ films showed good antimicrobial properties against both Gram-positive Staphylococcus aureus and Gram-negative bacteria Escherichia coli. Mucoadhesive properties of these films with respect to freshly excised sheep vaginal mucosa were evaluated using a tensile method. It was established that all films were mucoadhesive, but an increase in POZ content in the blend resulted in a gradual reduction of their ability to stick to vaginal mucosa. These films could potentially find applications in vaginal drug delivery.

## 1. Introduction

Vaginal drug administration has traditionally been used for the delivery of contraceptive agents and hormones as well as for local therapy of infections [1]. The vaginal route has several advantages, including the possibility to avoid first-pass metabolism, ease of administration and high permeability for small molecules.

The dosage forms traditionally used for vaginal drug delivery include creams, gels, pessaries, tablets, and elastomeric rings [2,3,4,5]. Mucoadhesive polymeric films have also received interest as a potential formulation strategy for vaginal delivery of contraceptives, microbicides and antimicrobial agents [6].

All water-soluble polymers have some ability to adhere to mucosal tissues, i.e., they exhibit mucoadhesive properties [7,8]. Typically, charged polymers of higher molecular weight show greater ability to adhere to mucosal membranes compared to non-ionic and smaller macromolecules. When weak anionic polyelectrolytes such as poly(carboxylic acids) are used, their mucoadhesive properties are related to hydrogen bonding with mucins [9]. Cationic polymers have excellent mucoadhesive properties due to electrostatic interactions with anionic mucin [10,11,12].

Chitosan is a cationic polysaccharide that exhibits excellent mucoadhesive properties [13] and antimicrobial activity [14]. It has been widely used in the design of various formulations for transmucosal drug delivery. Some attempts were also reported on the modulation of mucoadhesive and other physicochemical properties of chitosan through its chemical derivatisation [15].

Some modulation in the properties of chitosan could also be achieved through simple blending with other non-ionic water-soluble polymers. Previously, blending of chitosan with some cellulose ethers has been used to modify mechanical and mucoadhesive properties of polymeric films for buccal drug delivery [16]. Blends of chitosan with poly(N-vinyl pyrrolidone) [17,18,19,20], poly(ethylene oxide) [17,19] and poly(vinyl alcohol) [17,21,22] were also studied extensively.

Poly(2-oxazolines) is an emerging class of polymeric materials that have found numerous biomedical applications [23,24]. Poly(2-ethyl-2-oxazoline) is one of the representatives of poly(2-oxazolines) family that is a non-ionic water-soluble polymer available commercially. The application of this material in the design of dosage forms for drug delivery has received a substantial interest in the last few years and it is often viewed as a potential alternative pharmaceutical excipient to well-established water-soluble polymers [25,26,27,28].

Recently, we reported the preparation of chitosan/poly(2-ethyl-2-oxazoline) films for application in ocular drug delivery [29]. The structure and physicochemical properties of these films were evaluated using Fourier-transformed infrared spectroscopy, thermal gravimetric analysis, differential scanning calorimetry, wide-angle X-ray diffraction, tensile testing and scanning electron microscopy. These studies indicated a complete miscibility between the polymers in the blends. The films were evaluated as potential dosage forms for ocular drug delivery both in vitro and in vivo.

In this study, we report the preparation of chitosan/poly(2-ethyl-2-oxazoline) films loaded with ciprofloxacin as a model drug. In vitro drug release studies using a Franz diffusion cell were conducted. Antibacterial activity of drug-free and drug-loaded films against both Escherichia coli and Staphylococcus aureus was evaluated. Adhesion of the films to freshly excised sheep vaginal mucosa was studied using a tensile test. 

## 2. Materials and Methods

### 2.1. Materials

Chitosan (CHI, M_W_ ~ 310–375 kDa with a degree of deacetylation of 75–85%), poly(2-ethyl-2-oxazoline) (POZ, M_W_ ~ 50 kDa and PDI 3–4), hydrochloric acid solution (HCl, 1 M), phosphate-buffered saline (PBS) tablets pH 7.4, ciprofloxacin (CF), bovine serum albumin, acetic acid, lactic acid, glucose and urea were purchased from Sigma-Aldrich (Gillingham, UK). A dialysis cellulose membrane tube (molecular weight cut-off 14 kDa) was purchased from Sigma-Aldrich (Gillingham, UK). All other chemicals were of analytical grade and used without further purification.

### 2.2. Preparation of Films

Polymeric films based on chitosan (CHI) and its blends with poly(2-ethyl-2-oxazoline) (POZ) were cast by the solvent evaporation method according to a protocol previously reported by our group with minor modifications [29]. 1% *w/v* aqueous solutions of CHI and POZ were prepared by dissolving pre-weighed amount of dry polymers at room temperature. CHI solution (pH ~ 3.8) was prepared in 0.1 M HCl by stirring magnetically for 12 h prior to casting. POZ solutions (pH ~ 6.8) were prepared in deionised water and allowed to stir continuously for 1 h. The prepared polymer solutions were mixed at different volume ratios and named as CHI (100), CHI/POZ: (80:20), (60:40) and (40:60). The pH of the combined solutions was in the range of 3.9–4.0. CHI/POZ solutions were magnetically agitated for 3 h until total homogeneous mixture was formed. Subsequently, each polymer blend (5 mL) was poured into 35 mm plastic Petri dishes and dried at room temperature for several days.

### 2.3. Preparation of Ciprofloxacin-Loaded Films

A stock solution of ciprofloxacin hydrochloride (10 mg/mL) was prepared by first dissolving 0.1 g of ciprofloxacin in 0.4 mL of 1 M HCl, before making the total volume to 10 mL. Then, 0.5 mL of ciprofloxacin hydrochloride solution was aspirated and added to 4.5 mL of each CHI and CHI/POZ solutions followed by stirring for 2 h, to make the final 0.1% *w/v* CF in polymer blends. Afterwards, prepared solutions were cast and dried as mentioned above. The content of ciprofloxacin in each film of 35 mm in diameter was 5 mg.

### 2.4. Characterisation of Films

#### 2.4.1. Film Thickness and Weight

Physical measurements such as film thickness and weight were determined according to a previously described protocol [30]. Three film samples with a diameter of 35 mm from each formulation were individually weighed using an analytical balance. The thickness of the films was measured at five randomly selected positions including the middle part using a digital calliper. The mean ± standard deviation values were calculated and are shown in Table 1.

#### 2.4.2. Folding Endurance

Three samples from each film formulation were cut into 2 × 2 cm squares. The folding endurance was determined according to the methodology reported in [31] by repeatedly folding the film at 180° longitudinally at the same place until breakage. The film exhibiting folding endurance value ≥300 without breaking is considered to have excellent flexibility.

#### 2.4.3. Surface pH Measurements

Each film formulation was cut into discs with the diameter of 10 mm, then placed in plastic Petri dishes (40 mm in diameter) and allowed to swell in contact with 1.5 mL of distilled water at room temperature for 30 min. Surface pH was measured using a glass electrode 781 pH/Ion Meter placed on the surface of the swollen films. Each sample was analysed three times and the mean values ± standard deviations were calculated.

#### 2.4.4. Transparency

The transparency of the films was measured using an Analytik Jena Specord^®^ 200 Plus UV/Vis spectrophotometer (Jena, Germany) to determine the percentage of light transmittance for each sample (1 × 4 cm strips) at two different wavelengths (400 and 600 nm) of visible light.

### 2.5. Antimicrobial Studies

Antibacterial activity of CHI and CHI/POZ films with and without ciprofloxacin (CF) was examined against two model microorganisms such as Escherichia coli ATCC 8739 (Gram-negative bacteria) and Staphylococcus aureus ATCC 6538-p (Gram-positive bacteria) using disc diffusion method [32]. Briefly, samples were exposed to bacteria on solid media (nutrient agar) and inhibition zone around each sample was measured and recorded. First, active colonies of a number of microorganisms were cultured. These microorganisms were then diluted using 5 mL saline solution (0.9% NaCl) until a colony count can be achieved which has the turbidity values of 0.5 a.u. equivalent to that of a McFarland standard solution (1.5 ∙ 10^8^ CFU/mL). Then, bacterial inoculum (1.5–2.0 mL) was laid over the nutrient agar plate using a sterile cotton swab. Film samples (6 mm diameter discs) were placed on the agar plate using sterile forceps and incubated for 24 h at 37 °C. Inhibition zone for bacterial growth was measured to estimate its inhibitory effects (Table 2). A disc with pure antibiotic (CF) was used as a control sample.

### 2.6. In Vitro Drug Release Experiments

Release of CF from films was carried out using a dialysis method with Franz diffusion cell (FDC) under “sink conditions”. The cellulose membrane was used as a barrier and placed between the donor and receptor compartments of FDC. The experiment was conducted using phosphate-buffered saline (PBS, pH = 7.4) as a medium solution. The volume of PBS in the receiving chamber of the cell was 30 mL, which was stirred at 80 rpm and maintained at 37 °C throughout the experiment. Dry films with CF were placed directly on dialysis membrane in the donor compartment of FDC without any previous wetting and 1 mL of aliquots were taken from the receptor compartment at predetermined time intervals and replaced each time with 1 mL fresh medium (PBS) to maintain a constant volume. All release experiments were carried out during 8 h. Three replicates were performed for each type of films.

The amount of released CF was determined using spectrophotometric technique at 272 nm with the help of an Analytik Jena Specord^®^ 200 Plus UV/Vis spectrophotometer (Jena, Germany). The percentage of drug released at each time point was calculated using a calibration curve (Figure S1 in Supplementary Information, R^2^ = 0.9999).

### 2.7. Ex vivo Mucoadhesion Studies on Sheep Vaginal Tissue

The adhesiveness of the films to vaginal mucosa was studied using a tensile method with Texture Analyser XT Plus (Stable Micro Systems Ltd., UK) equipped with a cylindrical aluminium probe P/25 (25 mm in diameter). During testing, each film was cut into spherically shaped discs (10 mm in diameter), and then were attached to the probe with the help of double-sided adhesive tape, which was secured to the mobile arm of the texture analyser. Isolated sheep vaginal tissues were obtained from Altyn-Orda Abattoirs (Almaty, Kazakhstan) immediately after animal slaughter, frozen and were transported to the laboratory in a polystyrene container. The mucosal membranes were subsequently defrosted upon arrival and carefully dissected using a sharp blade, avoiding contact with the internal mucosa. Each mucosal tissue was affixed securely on the mucoadhesion rig and was moisturised with simulated vaginal fluid (SVF) prior to each testing. SVF was prepared according to the previous literature report [33] with the following composition: NaCl 3.51 g/L; KOH 1.40 g/L; Ca(OH)_2_ 0.222 g/L; bovine serum albumin 0.018 g/L; lactic acid 2.00 g/L; acetic acid 1.00 g/L; glycerol 0.16 g/L; urea 0.40 g/L; glucose 5.0 g/L. The pH of SVF was adjusted to 4.2 using 1 M HCl.

During the adhesion tests, each film sample attached to the cylindrical probe was pressed onto the moist vaginal surface at a speed rate of 0.05 cm/s and 0.981 N and remained in contact for 30 s to ensure complete attachment. Then the probe was withdrawn at a speed rate of 0.05 cm/s and 0.001 N trigger force until complete detachment from the biological substrate. Data acquired from the detachment experiments were then used to evaluate the mucoadhesion strength, i.e., the maximum force required for the detachment (F_adh_) and the total work of adhesion (the area under the force/distance curve, W_adh_) values (Figures S2 and S3 in Supplementary Information). All measurements were conducted five times for each film sample.

### 2.8. Statistical Analysis

Data obtained during these experiments, i.e., the mean values and standard deviations were calculated and compared for differences using two-tailed Student’s *t*-test with GraphPad Prism statistical analysis software (GraphPad Software Inc., version 7.0; San Diego, CA, USA), where *p* < 0.05 was used as a statistically significant criterion.

## 3. Results and Discussion

### 3.1. Preparation and Characterisation of Films

Ciprofloxacin (CF) was used in the present work as a model antibiotic. In some sources, it is recommended as a therapeutic agent for the treatment of some vaginal infections [34]. Two types of samples were prepared: drug-free and CF-loaded films. Initially, all these films were evaluated for their physicochemical characteristics and properties such as thickness and weight, folding endurance, optical transparency, and surface pH (Table 1). All films had thicknesses in the range of 0.05 to 0.08 mm. Drug loading did not make any substantial effects on the sample thicknesses.

The flexibility of a polymeric material is important when considering that the films will be administered intravaginally and this should not result in a breakage. One of the methods to assess film flexibility is the evaluation of its folding endurance, i.e., the number of times the sample could be folded at the same place without breaking. According to the literature [35], the films exhibiting folding endurance value greater than 300 are considered to have excellent flexibility. Our results indicated excellent flexibility for CHI (100) and CHI/POZ (80:20) films (both drug-free and drug-loaded), with each formulation remaining intact after >300 repeating folds (Table 1). Folding endurance was found to be highest for CHI (100) (1300 ± 8) and lowest for CHI/POZ (40:60) (65 ± 5). Thus, an increase in the POZ content in the film resulted in a decrease in the folding endurance.

All drug-free films were homogeneous, transparent and smooth, which is in good agreement with our previous study demonstrating complete miscibility between these two polymers [29]. However, the films containing ciprofloxacin were slightly opaque.

Transparency of the films was evaluated by measuring the light transmittance using UV/Vis-spectrophotometry at two wavelengths of visible light (400 and 600 nm). Almost all the drug-free films showed light transmission values above 85%, confirming their good transparency. Drug-loaded films exhibited much lower transparency with the light transmission values at 35.9–45.4% at 400 nm and 65.1–78.2% at 600 nm. An insufficient transparency of drug-loaded films could be a serious limitation in certain therapeutic areas, for example, in ocular drug delivery where it could interfere with normal vision. However, vaginal administration does not have a requirement for a dosage form to have particular transparency. The reduced transparency of drug-loaded films indicates that CF content in the samples exceeds its intrinsic solubility in these polymers in the solid state, resulting in its partial crystallisation.

The measurements of the samples’ surface pH established that these materials have an acidic nature, with drug-free films exhibiting pH ~ 4.02–4.14 and drug-loaded films showing slightly lower values (pH ~ 3.76–3.86). This acidic nature of the films makes them suitable for vaginal administration as the pH in a healthy adult vagina is also weakly acidic [36]. It may be concluded that vaginal pH will remain unaffected after administration of these films.

### 3.2. Antimicrobial Activity

Due to its cationic nature, chitosan is known to exhibit good antimicrobial activity against a wide range of microorganisms, such as bacteria, fungi and yeast [14,37,38]. However, these properties of CHI are greatly dependent on its physicochemical characteristics such as molecular weight, degree of deacetylation as well as biopolymer concentration, and environmental pH.

**Figure 1:**
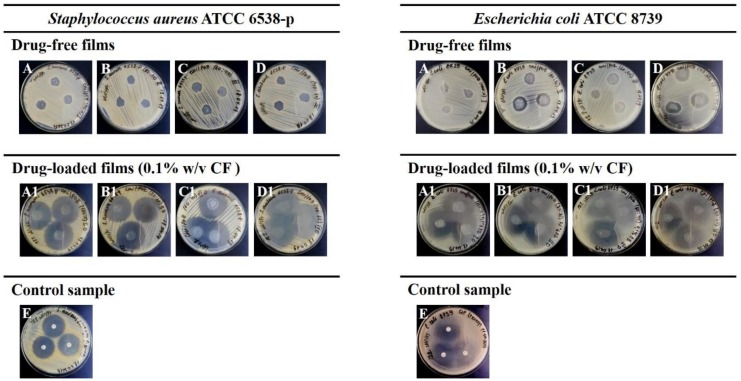
Inhibition zones of polymeric films against Gram-positive Staphylococcus aureus and Gram-negative Escherichia coli bacteria: **A** and **A1—**CHI (100); **B** and **B1—**CHI/POZ (80:20); **C** and **C1—**CHI/POZ (60:40); **D** and **D1—**CHI/POZ (40:60); and **E—**ciprofloxacin.

Antimicrobial activity of CHI and CHI/POZ films was studied against Staphylococcus aureus and Escherichia coli using the disc diffusion method. Figure 1 shows exemplar images from disc diffusion experiments and Table 2 summarises the data on the diameters of inhibition zones. It can be seen from these data that there is no suppression of the growth of Staphylococcus aureus strain for the film samples based on chitosan and poly(2-ethyl-2-oxazoline) without ciprofloxacin. Drug-free films of pure CHI also did not show any antimicrobial effects on Escherichia coli; however, CHI/POZ blends exhibited some inhibition in the growth of these bacteria. Several reasons could be responsible for lack of antimicrobial activity exhibited by the films of pure CHI to both Staphylococcus aureus and Escherichia coli, and also of the polymer blend films to Staphylococcus aureus. First, it could be related to the nature of the disc diffusion method and inability of high-molecular CHI (M_W_ ~ 310–375 kDa) to diffuse through agar gel and inhibit the growth of bacteria. Semi-crystalline nature of CHI also makes it insoluble at higher pHs and less diffusive. However, in the blends chitosan may be less crystalline and could diffuse better. Second factor is the sensitivity of different bacteria to antimicrobials and also to pH. Perhaps, Escherichia coli is more sensitive than Staphylococcus aureus either to chitosan or to lower pHs, displayed by the films.

CF was used as a control sample in these microbiological experiments and demonstrated antimicrobial activity against both Staphylococcus aureus ATCC 6538-p (growth inhibition zone of 31.2 ± 0.4 mm) and Escherichia coli ATCC 8739 (growth inhibition zone of 35.1 ± 1.2 mm), which characterises these strains as sensitive to this antibiotic.

Ciprofloxacin-loaded (0.1% w/v) polymeric films with CHI 100 (A1), CHI/POZ (80:20) (B1), CHI/POZ (60:40) (C1) and CHI/POZ (40:60) (D1) were active against Staphylococcus aureus ATCC 6538-p and growth inhibition zones were in the range from 36.0 ± 1.9 mm to 46.1 ± 1.7 mm. CHI/POZ films with ciprofloxacin also showed activity against Escherichia coli ATCC 8739 and displayed growth-inhibition zones in the range from 39.3 ± 2.3 mm to 42.5 ± 2.0 mm, which indicates greater antimicrobial activity against this bacteria strain. Thus, using CHI/POZ films as excipients for formulating CF results in enhancement of their antimicrobial activity against Gram-negative strain Escherichia coli in comparison with pure CHI film and control sample of CF. Sensitivity to the Gram-positive strain, Staphylococcus aureus, was observed only for drug-loaded films.

### 3.3. In vitro Drug Release Studies

**Figure 2:**
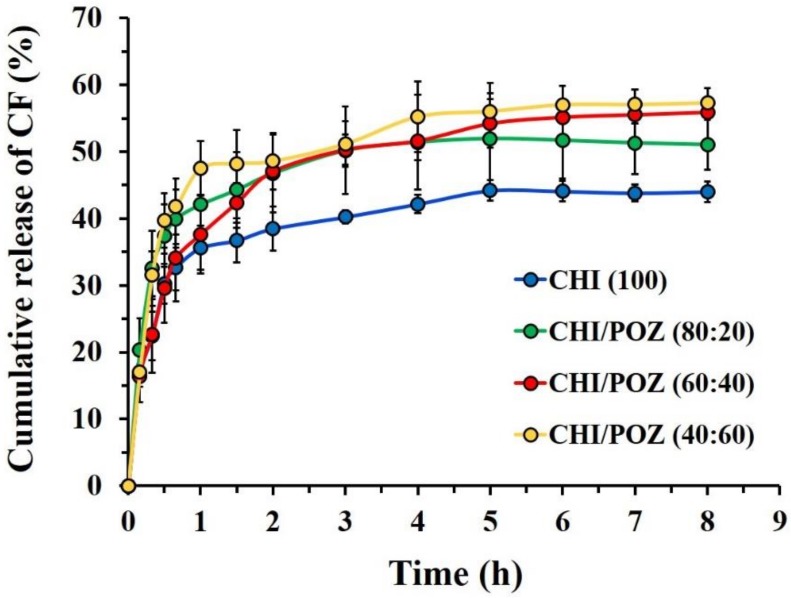
In vitro cumulative release of ciprofloxacin from chitosan (CHI) and chitosan/poly(2-ethyl-2-oxazoline) (CHI/POZ) films with different polymer ratios. Data are presented as mean ± standard deviation (n = 3).

The normal pH in the vagina of a healthy women of reproductive age is typically ranged within 3.8–4.2 [39]. This weakly acidic environment is due to lactic acid produced by the healthy vaginal microflora. However, in pathological vaginal conditions (e.g., bacterial vaginosis, trichomonas vaginalis, group B streptococcus or other pathogenic organisms), the pH may increase [40]. In order to model the vaginal environment with a pathological condition, the in vitro drug release studies from CHI/POZ films were conducted in PBS solution at pH = 7.4 (37 °C) using a dialysis membrane and the cumulative release profiles were calculated (Figure 2). CHI films showed the lowest drug release (no more than 45%), which is possibly due to the electrostatic attraction between the amino-groups of CHI and carboxylic groups of ciprofloxacin hydrochloride. The presence of poly(2-ethyl-2-oxazoline) in CHI/POZ films from 20% to 60% *v/v* resulted in an increase in drug released in the range of 51 ± 3% to 56 ± 2% during the 4 h, respectively. In our previous study [29], we confirmed the formation of weak intermolecular hydrogen bonds between carbonyl groups of poly(2-ethyl-2-oxazoline) and both amine and hydroxyl groups of chitosan. Thus, the presence of poly(2-ethyl-2-oxazoline) in the blend films can reduce the concentration of chitosan, which eventually leads to less efficient binding of CF and as a result, the amount of released drug is higher for CHI/POZ films in comparison with pure CHI. This electrostatic binding between ciprofloxacin and chitosan could also be the reason for an incomplete drug release from the films (only 40–55%). For all types of films, the drug release reaches the equilibrium value within approximately two hours.

### 3.4. Ex Vivo Mucoadhesion Studies

**Figure 3:**
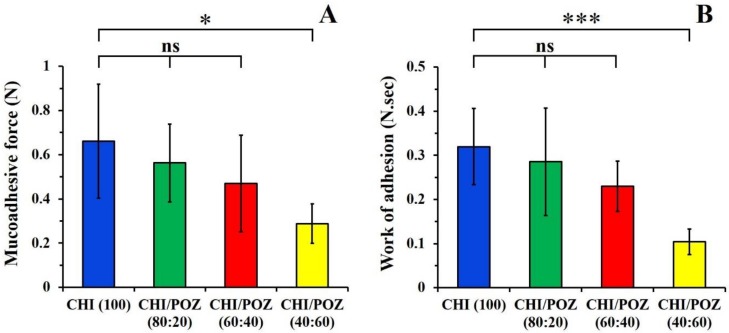
Detachment force F_adh_ (**A**) and total work of adhesion W_adh_ (**B**) values for detachment of chitosan (CHI) and chitosan/poly(2-ethyl-2-oxazoline) (CHI/POZ) blend films from sheep vaginal mucosa. Data are expressed as mean ± standard deviation (n = 5). Statistically significant differences are given as: *—*p < 0.05*; ***—*p < 0.001*; ns—no significance.

Mucoadhesive properties of films usually determine their residence on mucosal tissues. Films with good mucoadhesiveness are expected to retain on vaginal mucosa for a longer time and to maintain high drug levels at the site of administration [41]. The tensile method is one of the approaches widely used to evaluate mucoadhesive properties of various formulations, including films [42]. In this work, we have used freshly excised sheep vaginal tissues as a substrate for mucoadhesion studies. The values of maximum detachment force (F_adh_) and the total work of adhesion (W_adh_) for detachment of drug-free CHI and CHI/POZ films from vaginal tissue were determined (Figure 3).

Films based on pure chitosan exhibit strong mucoadhesive properties due to its cationic nature and electrostatic attraction to negatively charged mucosa. Additionally, its hydroxyl groups could also form hydrogen bonds with mucin [13]. On the contrary, poly(2-ethyl-2-oxazoline)s exhibit poor mucoadhesive properties [15,43]. The weak mucoadhesive nature of POZ can be explained by its non-ionic nature. An increase in POZ content in the films shows a gradual reduction in the detachment force and total work of adhesion values, which is consistent with the decrease in the concentration of more mucoadhesive component (chitosan). There is also a good correlation between F_adh_ and W_adh_ values. This trend is also in good agreement with our previous studies of mucoadhesive properties of chitosan blends with hydroxyethylcellulose (as a non-ionic component) with respect to porcine buccal mucosa [16].

Potentially, the films loaded with ciprofloxacin could also exhibit some reduction in their mucoadhesive properties compared to drug-free blends due to the inability of small drug molecules to contribute to adhesion. This trend was previously reported by us for chitosan tablets loaded with ibuprofen [44].

## 4. Conclusions

Polymer blends of chitosan and poly(2-ethyl-2-oxazoline) were prepared in the form of flexible and transparent films by casting of aqueous solutions with subsequent solvent evaporation. Samples of pure chitosan films without the addition of ciprofloxacin did not demonstrate antibacterial activity against Staphylococcus aureus and Escherichia coli. The CHI/POZ films showed some antimicrobial properties with respect to Escherichia coli, but did not show an ability to inhibit the growth of Staphylococcus aureus. Polymer films with CF showed antimicrobial activity against both strains of bacteria. Pure chitosan films with CF demonstrated the lowest drug release as a result of possible electrostatic attraction between the amino-groups of chitosan and carboxylic groups of ciprofloxacin hydrochloride. Films based on pure chitosan and its blends with poly(2-ethyl-2-oxazoline) exhibited mucoadhesive properties with respect to freshly excised sheep vaginal tissue. These formulations could potentially be used as mucoadhesive films for vaginal drug delivery.

## Figures and Tables

**Table 1:** Physicochemical parameters of chitosan (CHI) and chitosan/poly(2-ethyl-2-oxazoline) (CHI/POZ) blend films.

Samples	Chemical Composition (% v/v)	Thickness (mm)	Weight (mg)	Folding Endurance	Transparency (%)		Surface pH
					400 nm	600 nm	
**Drug-Free Films**							
**A**	CHI 100	0.07 ± 0.01	33.41 ± 1.52	>300	85.6	89.8	4.06 ± 0.37
**B**	CHI/POZ 80:20	0.08 ± 0.02	33.67 ± 2.51	>300	87.5	89.4	4.02 ± 0.30
**C**	CHI/POZ 60:40	0.06 ± 0.01	35.00 ± 4.36	<300	88.2	89.9	4.08 ± 0.33
**D**	CHI/POZ 40:60	0.05 ± 0.01	33.33 ± 2.08	<300	88.5	89.7	4.14 ± 0.32
**Drug-Loaded Films**							
**A1**	CHI 100	0.06 ± 0.01	35.25 ± 2.29	>300	36.6	65.1	3.76 ± 0.49
**B1**	CHI/POZ 80:20	0.07 ± 0.01	32.70 ± 1.51	>300	45.4	68.1	3.86 ± 0.22
**C1**	CHI/POZ 60:40	0.06 ± 0.01	36.45 ± 5.31	<300	35.9	72.9	3.78 ± 0.42
**D1**	CHI/POZ 40:60	0.05 ± 0.01	38.83 ± 1.83	<300	40.5	78.2	3.86 ± 0.22

**Table 2:** Antimicrobial activity of CHI and CHI/POZ blend films.

Samples	Chemical Composition (% v/v)	Diameter of Growth Inhibition Zone (mm)	
		Staphylococcus aureus	Escherichia coli
**Drug-Free Films**			
**A**	CHI (100)	0	0
**B**	CHI/POZ (80:20)	0	17.3 ± 2.1***
**C**	CHI/POZ (60:40)	0	13.3 ± 2.1**
**D**	CHI/POZ (40:60)	0	21.0 ± 2.0****
**Drug-Loaded Films (CF 0.1% w/v)**			
**A1**	CHI (100)	38.6 ± 1.3***	39.5 ± 1.9*
**B1**	CHI/POZ (80:20)	36.0 ± 1.9**	40.1 ± 2.6**
**C1**	CHI/POZ (60:40)	38.6 ± 1.6***	42.5 ± 2.2***
**D1**	CHI/POZ (40:60)	46.1 ± 1.7***	39.3 ± 2.3*
**E**	Disc with CF	31.2 ± 0.4	35.1 ± 1.2

CHI, chitosan; POZ, poly(2-ethyl-2-oxazoline); CF, ciprofloxacin. Anti-microbial activity values of CHI/POZ drug-free films and ciprofloxacin-loaded films were compared to pure CHI and discs with CF, respectively. Statistically significant differences are given as: ****—*p < 0.0001*; ***—*p < 0.001*; **—*p < 0.01;* *—*p < 0.05*. Data are expressed as mean ± standard deviation (n = 3).

## Supplementary Materials

The following are available online at https://www.mdpi.com/1996-1944/13/7/1709/s1, Figure S1: A calibration curve used to determine the amount of released ciprofloxacin (CF) from chitosan based films, Figures S2 and S3: Exemplary detachment profiles of CHI (100) and CHI/POZ (40:60) films.
