# Supplementary material for: Chitosan/Poly(2-ethyl-2-oxazoline) Films with Ciprofloxacin for Application in Vaginal Drug Delivery
Source: Materials (Basel). 2020 Apr 6;13(7):1709. doi: 10.3390/ma13071709 (PMC7178655; doi:10.3390/ma13071709)
Supplement: Supplementary file 1 [file materials-13-01709-s001.pdf]

Supplementary Information

Chitosan/poly(2-ethyl-2-oxazoline) mucoadhesive films with ciprofloxacin for application in vaginal drug delivery

Guzel K. Abilova[1,2], Daulet B. Kaldybekov[1], Galiya S. Irmukhametova[1], Diara S. Kazybayeva[1], Zhanar A. Iskakbayeva[3], Sarkyt E. Kudaibergenov[4], Vitaliy V. Khutoryanskiy[5,*]

[1] Department of Chemistry and Chemical Technology, Al-Farabi Kazakh National University, 050040 Almaty, Kazakhstan; guzelab82@mail.ru (G.K.A.); dauletchem@gmail.com (D.B.K.); galiya.irm@gmail.com (G.S.I.); diara_92@mail.ru (D.S.K.)

[2] K. Zhubanov Aktobe Regional State University, 030000 Aktobe, Kazakhstan

[3] Microbiology Laboratory of the Scientific Center for Anti-Infectious Drugs, 050060 Almaty, Kazakhstan; (Zh.A.I.); zhanara_07_74@mail.ru

[4] Institute of Polymer Materials and Technologies, 050013 Almaty, Kazakhstan; skudai@mail.ru (S.E.K.)

[5] Reading School of Pharmacy, University of Reading, Whiteknights, RG6 6AD Reading, United Kingdom

* Correspondence: v.khutoryanskiy@reading.ac.uk (V.V.K.)

***Corresponding author:**

Postal address: Reading School of Pharmacy, University of Reading, Whiteknights, PO Box 224, RG6 6AD Reading, United Kingdom

E-mail address: v.khutoryanskiy@reading.ac.uk (Prof V.V. Khutoryanskiy)

Telephone: +44(0) 118 378 6119

Fax: +44(0) 118 378 4703

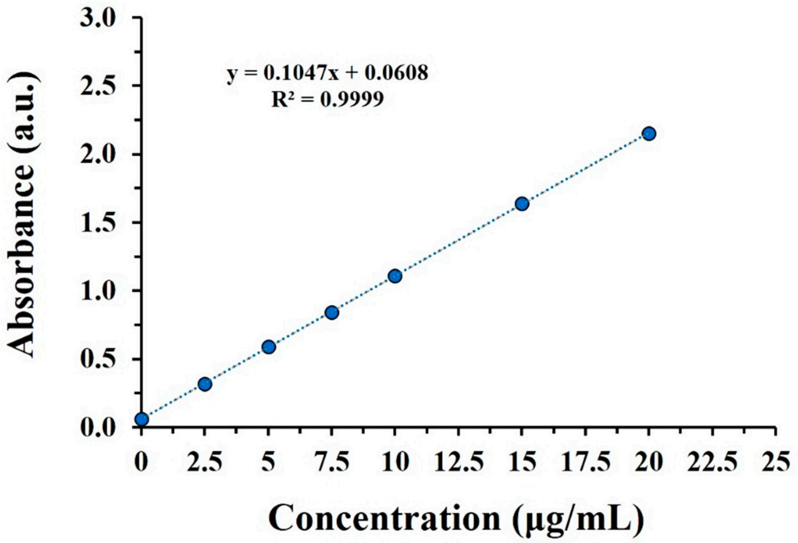

Figure S1. Calibration curve used to determine the amount of ciprofloxacin released from CHI and CHI/POZ films.

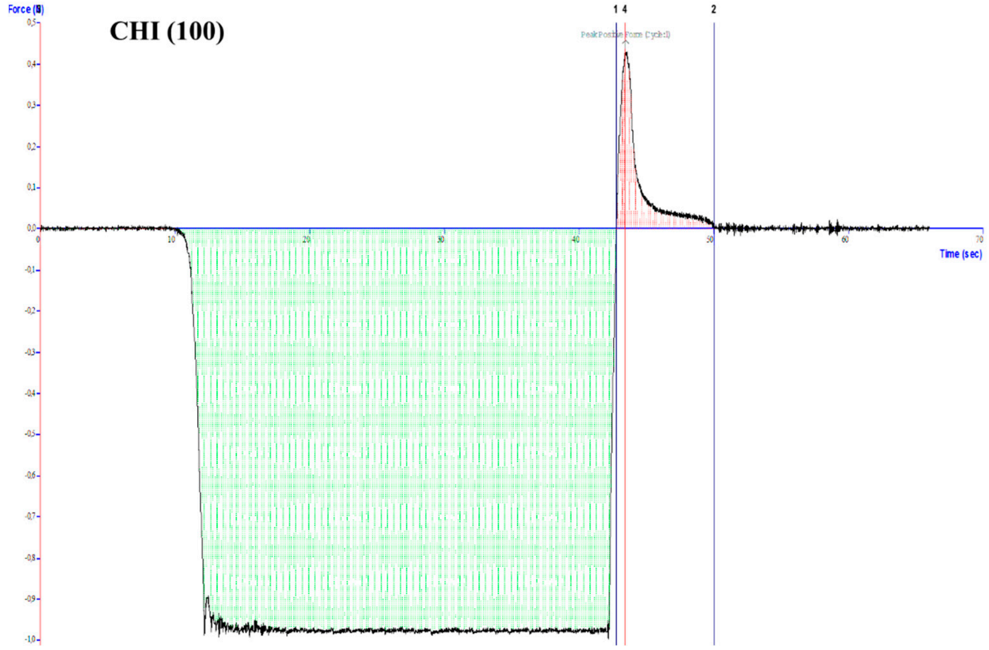

Figure S2. Exemplar detachment profile of pure chitosan (CHI) films from sheep vaginal mucosa.

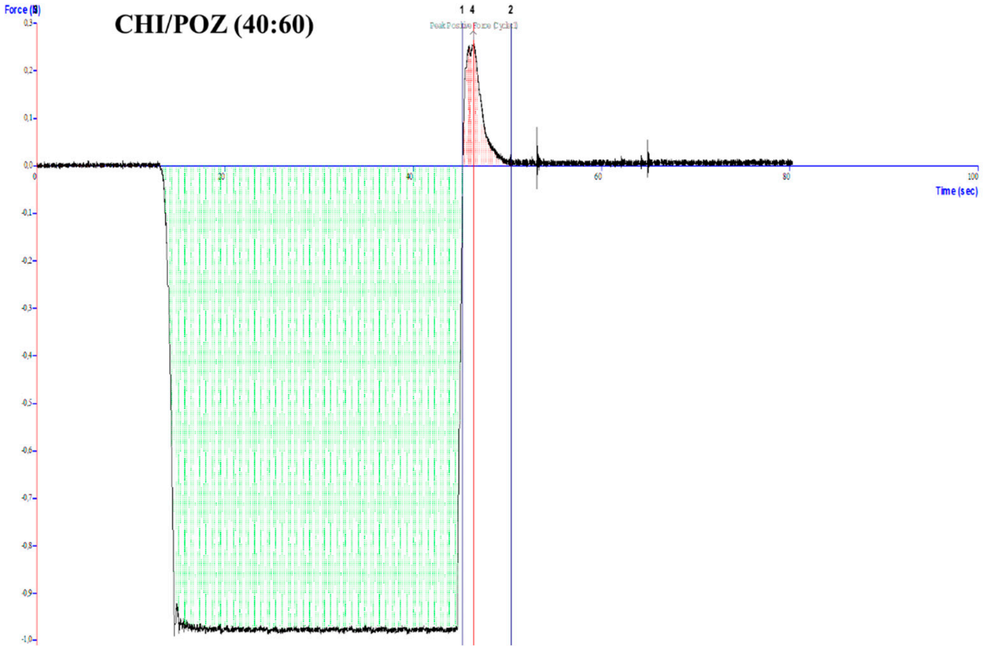

Figure S3. Exemplar detachment profile of pure chitosan–poly(2-ethyl-2-oxazoline) (40:60) films from sheep vaginal mucosa.
